# Using treatment guidelines to improve antibiotic use: insights from an antibiotic point prevalence survey in Kenya

**DOI:** 10.1136/bmjgh-2020-003836

**Published:** 2021-01-08

**Authors:** Michuki Maina, Jacob McKnight, Olga Tosas-Auguet, Constance Schultsz, Mike English

**Affiliations:** 1 Health Services Unit, KEMRI-Wellcome Trust Research Programme Nairobi, Nairobi, Nairobi, Kenya; 2 Amsterdam UMC, Department of Global Health, University of Amsterdam, Amsterdam, The Netherlands; 3 Nuffield Department of Medicine, University of Oxford, Oxford, Oxfordshire, UK; 4 Amsterdam Institute for Global Health and Development, Amsterdam, The Netherlands

**Keywords:** treatment, health policy, health systems, public health

**Summary box**

- Clinical practice guidelines have the potential to improve quality of care through improving decision making and antibiotic prescription. These guidelines are particularly important in areas with limited laboratory and specialist capacity.
- For some of the common conditions managed in the hospitals, guidelines are not available or are outdated.
- To reduce irrational antibiotic use and contain the threat of antimicrobial resistance, the process of guideline development should prioritise the most common diseases.
- The process of developing context-appropriate clinical guidelines requires input from all relevant stakeholders with leadership from the Ministry of Health. This process needs to have a clear plan for dissemination, training and future updates.

## Background

Antimicrobial resistance (AMR) is a significant public health threat that is expected to worsen as more drug-resistant organisms emerge.^1^ This situation is further exacerbated by the low rate of discovery of new antimicrobial agents that could act against drug-resistant micro-organisms. AMR could retard economic growth in low-income countries and delay attainment of the sustainable development goals.^2^

There are multiple drivers of AMR, but one of the key drivers has been the irrational use of antimicrobial agents.^3^ In hospitals, lack of timely and accurate diagnostic tests, including microbiology for bacterial speciation and drug susceptibility testing, leads to unnecessary antimicrobial use, fueling resistance and healthcare costs.^4^ While countries must work to improve diagnostic capabilities and increase laboratory capacity to enhance diagnostic accuracy, it is also important to complement this new capacity with locally relevant guidelines. Providing context-specific, applicable and regularly updated treatment guidelines to front-line doctors is an effective means to improve antibiotic usage and clinical care.^5^

**Figure 1:**
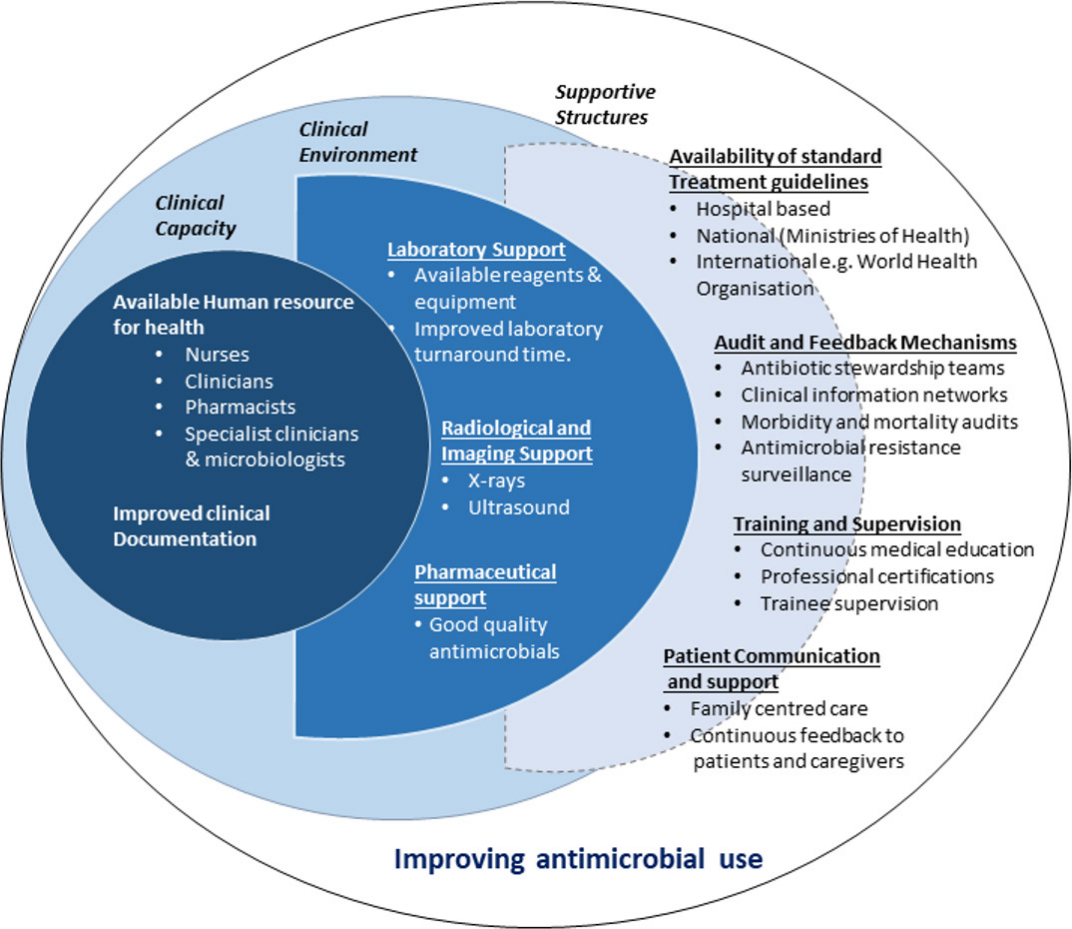
Improving antimicrobial usage requires clinicians, laboratory and radiological support in an environment that provides treatment guidelines, training, feedback and patient communication.

Clinical practice guidelines (CPG) provide a standardised and systematic approach to responding to disease, including the treatment. The guidelines are, however, more effective in the context of a functioning health system with adequate clinicians, drugs, diagnostics and a supportive environment to the clinicians and patients.^6^ This multifaceted approach of improving antimicrobial usage is illustrated in figure 1.

Here, we present data from an antibiotic point prevalence survey that illustrates the continued importance of developing guidelines for improving diagnosis and treatment. We propose that guidelines should be developed to target common diseases in limited-resource settings as a priority, and lastly, we illustrate how guideline development and dissemination at scale can be achieved using the example of Kenya’s basic paediatric protocols (BPP).

## Using guidelines to improve diagnosis and antibiotic use

A point prevalence survey was conducted across 14 public county hospitals (formerly district hospitals) in Kenya. These hospitals, with varying bed capacities, are located in urban and rural areas of Kenya with high and low malaria endemicity. They provide multispecialty inpatient care, which includes; maternal, neonatal, adult and paediatric medical and surgical units. Data from this survey conducted among hospitalised patients revealed that large proportions of patients received antibiotic treatment for conditions that did not warrant antibiotics.^7^ However, this inappropriate use of antibiotics was much less prevalent in the paediatric medical (14% (36/261) and neonatal units (5% (11/224)) where treatment guidelines were physically available than on adult medical units where treatment guidelines were absent, and 33% (140/421) of the patients were inappropriately treated.

In addition to lack of guidelines, the literature on irrational antibiotic use suggests that the level of training of the prescribers, the fear of the clinicians missing an existing infection, fear of lawsuits, fear of being reprimanded by more senior colleagues and pressures from patients, nurses and other ward staff can especially encourage overtreatment.^8 9^ Availing standard guidelines can help address many of these factors and give confidence to the clinicians on what comprises best practice while also improving the accuracy of diagnoses and treatment.^10^

## The need for guidelines

While it may be ideal to have guidelines for all diseases, immediate efforts should be directed to the most common ailments. On the neonatal and paediatric units we examined, the CPG available in the wards^11^ provided treatment advice that spanned 94% (211/224) and 55% (143/261) of the admissions, respectively. There were guidelines for common conditions in adults or on surgical units.

The main adult conditions for which antibiotics were prescribed among the adult medical and surgical populations included; pneumonia, obstetric and gynaecological infections, HIV-associated infections, central nervous system (CNS) infections, skin and soft-tissue infections (SSTI), and antibiotics for surgical prophylaxis. In the paediatric medical unit, the common conditions included; pneumonia, CNS infections, gastrointestinal infections and sepsis.^7^ Here, we use the example of pneumonia and CNS infections in adults and SSTI in surgical units to illustrate the need for guidelines.

In the adult medical wards, pneumonia and CNS infections accounted for 22% (94/421) and 20% (50/421) of admissions, respectively. Based on predefined criteria, 26% and 28% of these patients admitted with pneumonia and CNS infections, respectively, received inappropriate treatment. In the surgical units, SSTI which lack current local guidelines were a common cause of hospitalisation in adults (25% (135/543)) and children (60% (32/53)). Of these patients with SSTI, the documented antibiotic treatment was inappropriate in 69% and 43% of the adults and children, respectively. The choice of antibiotics used to treat these skin infections varied widely across the hospitals surveyed. Additionally, there was a significantly higher use of nitroimidazole derivatives compared with the preferred beta-lactam antibiotics.^12^

Availing approved guidelines for these conditions could ensure their treatment is standardised across hospitals. To reduce AMR, these guidelines should be in line with the recommendations by the WHO essential medicines list that encourages the use of the access group drugs (generally having a narrow spectrum of activity) as first-line and second-line therapy under the Access, Watch and Reserve categorisation.^13^ Treatment guidelines for adults in medical and surgical units which cover these, and other common conditions were developed in Kenya. However, they have not been updated for 12 years; they were not disseminated in easy to use formats or at scale and were not found in any of the hospitals visited.^14^ Therefore, it is essential to update or replace these guidelines in a format that can be widely and rapidly disseminated. It would be advisable to draw on international guidelines, including those from WHO^15–17^ and include context-specific modifications.^18^ This process should be driven from the ‘bottom-up’ by the professional medical and surgical associations with strategic direction offered by the Ministry of Health as the Ministry is mandated to generate health policies.

## The potential for national guidelines

Unlike in high-income settings where guidelines may be hospital or region-specific, in many low-income and middle-income countries, guidelines tend to be for national use, including the private sector. Key to the development process, therefore, are the Ministries of Health who provide direction on what guidelines need to be developed based on the local disease burden. The ministry also plays a role in ensuring adequate representation by all relevant stakeholders in the development process that should be transparent and not undermined by conflicts of interest or undue influence from specific individuals or groups (including funders).^19^ Guideline development should specifically extend to include plans for training and dissemination activities targeting all relevant clinicians in preservice training or practice in the public, not-for-profit and private sectors. This includes availing the guidelines in electronic versions for higher utilisation.^20^

Other stakeholders, especially organisations such as WHO can play a vital role by providing access to high-quality evidence syntheses and offering training and technical support to the process of guideline development.^21^

## The Kenyan BPP as an example of a national guideline development process

Developing guidelines is a multidisciplinary effort that requires input from expert clinicians, representatives from professional bodies and end-users, economists and methodologists. These teams need to be responding to relevant clinical needs that require the development of guidelines.^21^

In Kenya, this approach was adopted to develop the BPP first published in 2006. The development of these guidelines was initiated and overseen by the Ministry of Health with input from the professional paediatric association, universities and clinicians.^22^ As the process evolved, topics were identified in response to clinician queries for conditions that lacked clear guidelines and systematic reviews were conducted to generate context-appropriate evidence. This evidence was presented to multiple stakeholders, and guidelines were then developed through consensus. The BPP underwent three updates in 2010, 2013 and 2016 to include new and emerging evidence.^11 23^

Guideline availability, however, does not necessarily translate into their use. It is, therefore, essential to include a plan for training and implementation as part of the guideline development process.^23^ The development of the BPP was accompanied by the roll-out of the BPP-based Emergency Triage Assessment and Treatment Plus Admission training to clinicians in public and private hospitals and medical students. Many thousands of health workers and students have been trained since the guidelines were first rolled out, and numerous low-cost, short guidelines booklets have been distributed.^23^ Since their introduction, better case definition and management of pneumonia and diarrhoeal diseases, improved clinical documentation and a decline in the use of inappropriate cough medications have been reported among other benefits.^10 24 25^

## Conclusion

Availing up to date treatment guidelines to clinicians provides an opportunity to reduce inappropriate antibiotic use in hospitals. There are apparent gaps in guideline development and availability, especially for common adult medical conditions and across all ages in surgical care. Guideline development can be used to build consensus across a broad spectrum of the clinical community on contextually appropriate treatments. Their development must be accompanied by clear and adequate dissemination strategies to ensure all the clinicians making decisions daily, understand the rationale for the recommended strategy and have access to guidance at the point of care.
